# Assessment of the Dose–Response Relationship between Meal Protein Content and Postprandial Thermogenesis: Effect of Sex and the Oral Contraceptive Pill

**DOI:** 10.3390/nu11071599

**Published:** 2019-07-15

**Authors:** Maharani R. Duhita, Yves Schutz, Jean-Pierre Montani, Abdul G. Dulloo, Jennifer L. Miles-Chan

**Affiliations:** 1 Department of Endocrinology, Metabolism and Cardiovascular System, Faculty of Sciences and Medicine, University of Fribourg, 1700 Fribourg, Switzerland; 2 Human Nutrition Unit, School of Biological Sciences, University of Auckland, 1010 Auckland, New Zealand

**Keywords:** dietary protein, energy metabolism, sex difference, oral contraceptive pill, thermogenesis, energy balance

## Abstract

Implementation of efficacious dietary interventions to regulate energy balance requires understanding of the determinants of individual response. To date, information regarding individual variability in response to elevated meal protein content is lacking. This study investigates whether sex and/or oral contraceptive pill (OCP) use play a role in the response to elevated meal protein in 21 healthy young adults (seven men, seven women not taking OCP, and seven women who were OCP users). Participants consumed each of three standardized isocaloric (590 kcal) meals of differing protein content (11, 23, 31% kcal protein). Resting energy expenditure (EE), respiratory quotient (RQ), hunger and satiety were measured at baseline (fasting) and during 180 min postprandial. Whilst significant dose–response increases in EE were observed in men, meal protein-induced EE in women without OCP reached a maximum at <23% protein. Women taking OCP reported lower postprandial fullness than women without OCP, despite similar body size, but also, most notably, no significant difference in EE response between any of the meals. Whilst the mechanisms underpinning this thermogenic inflexibility in response across a wide-range (three-fold) of protein meal content require further investigation, this highlights the need for careful consideration of factors that may influence an individual’s metabolic response to dietary interventions aimed at optimising postprandial thermogenesis for body weight regulation.

## 1. Introduction

In the search for dietary strategies to enhance thermogenesis for the management of obesity and obesity relapse (successful long-term weight maintenance), dietary protein has been a focus of much research. However, whilst increasing protein consumption has been shown to be somewhat effective in modulating energy balance at a macro-level [1,2], there appears to be a high degree of variability in individual responses to such interventions [3,4]. Indeed, in a recent study, we demonstrated no greater thermic effect of a high protein (24% total energy from protein) versus normal protein (11%) meal in women taking the combined, monophasic oral contraceptive pill (OCP) [5]. Although the mechanisms underpinning this differential response are not yet known, it should be noted that high protein diets administered in the context of weight management often include meals comprising about 30% of energy intake as protein, that is, higher than the 24% protein meal used in our above-mentioned study [5]. Combined with concerns about the long-term consequences of “too high” protein diets on kidney function and risks for cardiovascular diseases [6], there is therefore a need to determine whether or not the effect of contraceptive pill use on metabolic responsiveness to dietary protein persists at this higher level of meal protein content (i.e., 30% by energy), whether any sex-differences in response become apparent across a wider content range, and to establish the range of what could constitute an acceptable “high-protein” diet for weight management. Crucially, and perhaps surprisingly, to date there has been no comprehensive, laboratory-controlled study investigating the acute dose–response relationship between meal protein content and postprandial energy expenditure (EE) under isocaloric conditions. As interest into the effects of OCP on the thermogenic and satiety responses to dietary protein levels is novel, it is important to first describe its impact in healthy young lean subjects—which furthermore may have implications for the development and prevention of obesity.

Therefore the aim of the present study was to investigate the dose–response relationship between meal protein content, energy expenditure (EE), respiratory quotient (RQ; an index of substrate utilisation) and satiety in healthy young adults by investigating these metabolic responses to the ingestion of three standardized isocaloric diets of differing protein content (11, 23, and 31% kcal protein), particularly taking into account the role of sex and OCP use. We hypothesised that the thermogenic and satiety responses to the meal would increase in a stepwise fashion in response to increasing meal protein content in men and in women without OCP, but not in women using OCP. 

## 2. Materials and Methods 

### 2.1. Subjects

14 healthy, non-obese young women (7 not taking and 7 taking the combined, monophasic OCP), and 7 healthy, young men were recruited for this study. The number of required subjects was determined by power analysis using web-based software (http://clincalc.com/stats/samplesize.aspx) with a type I error (α) of 0.05 and a desired power (1 − β) of 0.90. Based on our previous work [5], to detect a difference in the area-under-curve (AUC) (normal–high protein) for EE between –OCP and +OCP of 0.05 kcal/min based on a SD of 0.04 kcal/min, a sample size of 7 subjects per group was required.

Exclusion criteria were as follows: regular smokers, claustrophobic, pregnant or breastfeeding women, subjects with acute infections, chronic inflammatory disease, a history of metabolic disease (e.g., diabetes), cardiovascular disease, neurological or gastro-intestinal disease, eating disorders or food allergies, irregular menstruation, or taking medication other than the OCP which could interfere with metabolic rate. All women in the OCP group had been taking a pill containing 26 ± 3 μg (mode: 20 μg) of ethinyl estradiol for at least 3 months, as per the manufacturer’s instructions. 

This study complied with the Declaration of Helsinki and was approved by the state ethical review board; all participants gave written consent. This study was registered within the ISRCTN registry prior to recruitment (ISRCTN57611296).

### 2.2. Experimental Design

After an initial screening visit to complete the consent process and a diet/lifestyle questionnaire, participants attended the laboratory for three experimental sessions, with at least a 2 day interval between sessions. Subjects were asked to avoid intense physical activity and to abstain from alcohol and caffeine-containing foods and beverages for 24 h before each test. On the day of testing, participants arrived at the laboratory at 8:00 am following a 12 h overnight fast. Testing was conducted in the follicular phase of the menstrual cycle in women not taking OCP, and the week following withdrawal bleeding in women taking the OCP. Body composition was determined using multifrequency bioimpedance analysis (InBody 720, Biospace Co., Ltd., Seoul, Korea). Measurements of oxygen consumption (VO_2_) and carbon dioxide elimination (VCO_2_) were carried out using an open-circuit indirect calorimeter equipped with a ventilated canopy (hood) system (Quark CPET, COSMED, Rome, Italy). As previously described [5], participants were seated comfortably in a car seat adapted for calorimetric monitoring, with metabolic measurements conducted for at least 30–40 min, after 15 min of rest; for each subject the mean of last 30 min was taken as the baseline (fasted) value. The ventilated hood was then removed while the subject ingested one of the three test meals described below, consumed within 10 min. The ventilated hood was replaced, and calorimetric monitoring was continued for a further 180 min. While not capturing the entire thermic response, this duration of measurement minimises discomfort of the subjects under canopy, and has been shown to highly correlate (r > 0.95) with 6 h meal-induced thermogenesis for a test meal of this caloric content [7]. Participants were permitted to watch a calm movie or a documentary during the metabolic measurements but were not permitted to consume any additional foods or beverages (including water).

### 2.3. Test Meals

During each visit, participants consumed one of three ~590 kcal isocaloric test meals of varying protein content (11, 23, or 31% total energy from protein). Meals consisted of a simple breakfast meal base (oats, ground almonds, yoghurt, double cream) to which a protein powder supplement (containing 4:1 ratio of casein to whey protein) was added to increase the protein content as necessary, while maintaining the ratio of fat to carbohydrate constant at 0.71, as detailed in Table 1. Whilst the ingredients used in these test meals differed from our previous study [5], the ratio of non-protein macronutrients and total energy content of the meals used here were matched to our previous study [5] to facilitate comparison of results. The order of the test meals was randomized using a simple, single-block randomization plan (http://www.randomization.com), and participants were blinded to the meal content. 

### 2.4. Visual Analog Scales

In order to assess postprandial satiety, measures of hunger and fullness were obtained by visual analog scale (VAS) rating at baseline and 60, 120, and 180 min postprandial, according to the methodology of Stubbs et al. [8,9]. Specifically, participants were asked to rate their fullness (i.e., “how full are you at this moment”; anchors: “not at all full” to “extremely full”), desire to eat (i.e., “how strong is your desire to eat at this moment?”; anchors: “not at all strong” to “extremely strong”), and prospective food consumption (i.e., “how much food do you think you could eat at this moment?”; anchors: “none” to “a large amount”). 

### 2.5. Data Analyses

EE was calculated according to the Weir equation [10], with urinary nitrogen excretion assumed to be at a fixed average value of 13 g/24 h for each subject, reflecting the rate of urinary nitrogen excretion in the post-absorptive state [11]. Note that even a potential error of 10% in the N excretion would not significantly affect resting EE for two reasons: (1) The protein correction has very small power in the calorimetric equation, as compared to carbohydrate (CHO) oxidation and fat oxidation; and (2) the respiratory quotient (RQ) of protein oxidation (0.83) is in the middle band of the physiological RQ extreme span (0.7 for fat to 1.0 for CHO). RQ was derived from the ratio between VO_2_ and VCO_2_ (i.e., VCO_2_/VO_2_). The min-by-min EE, RQ and HR data yielded were processed as means of 30 min during the baseline and post-prandial period. All data are presented as mean ± SEM unless otherwise stated. Statistical analyses by one-way analysis of variance (ANOVA) or repeated-measures ANOVA followed by Dunnett’s multiple comparison tests (versus baseline) or Bonferroni post-tests (between groups) were performed using the computer software STATISTIX version 8.0 (Analytical Software, St Paul, MN, USA). 

## 3. Results

### 3.1. Participant Characteristics

The age, anthropometry and body composition data for the three subject groups are provided in Table 2**.** There were no significant differences in any of these parameters between the women taking the OCP (+OCP) and those not taking the OCP (-OCP). To determine the effect of sex on these characteristics, we compared men to all women (*n* = 14), and to the –OCP group alone, and found no significant differences in terms of age. However, the men were, on average, taller and heavier (both *p* < 0.001), with greater BMI (*p* = 0.01) and fat-free mass (*p* < 0.001), and a lower %Fat (*p* < 0.005) than the women. 

### 3.2. Baseline (Fasted) EE

Whilst baseline (fasted) EE was significantly higher in men than women (*p* < 0.0001; Table 3), this difference was no longer observed when adjusted for fat-free mass (*p* = 0.51). Similarly, there was no effect of OCP use (*p* = 0.74) on baseline EE, nor any difference in baseline EE across experimental days. The mean intra-individual coefficient of variation (CV) values for baseline (fasted) EE measured across the three experimental days were less than 6% in all three groups, in line with the intra-individual CV often reported for basal metabolic rate (BMR) [12].

### 3.3. Postprandial EE 

**Figure 1:**
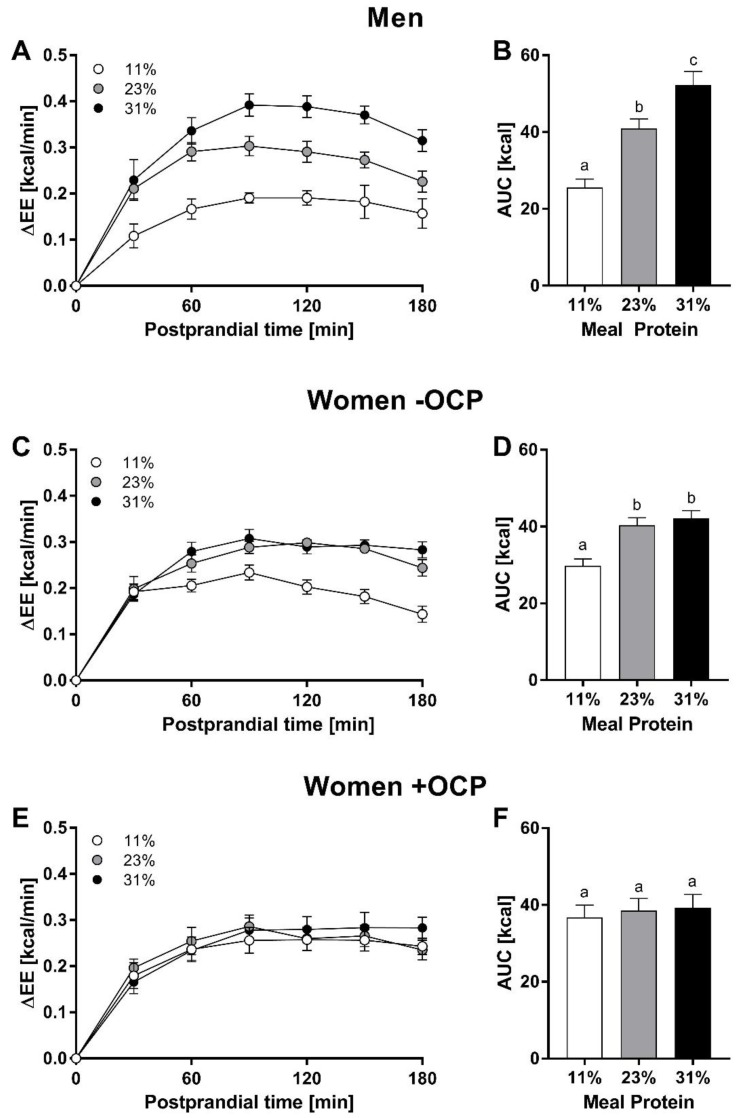
Change in resting EE (ΔEE) following ingestion of each of three isocaloric meals containing different protein levels (11, 23, and 31% total energy as protein). Values are mean ± SEM. Left panels (**A**,**C**,**E**) indicate 30 min means; right panels (**B**,**D**,**F**) indicate the area-under-curve (AUC) over 180 min. Bars not sharing superscript (i.e., a,b) are significantly different (*p* < 0.05) from one another by repeated measures ANOVA. OCP: oral contraceptive pill.

Figure 1 shows changes in resting EE (ΔEE) following ingestion of an isocaloric meal containing either 11, 23, or 31% of total energy as protein in a repeated measure design. A dose–response was observed in men, with significant stepwise increases in EE with increasing protein dose (Figure 1A,B; *p* < 0.05). In women not taking OCP (women -OCP group), the 23% protein meal significantly increased EE above the level observed following the 11% protein meal (Figure 1C and 1D; *p* < 0.001); however, no significant increase in EE between the 23% and the 31% protein meal was observed (*p* = 0.8). Interestingly, no effect of meal protein content was seen in women taking OCP (women +OCP group), with no significant difference in response between any of the test meals (Figure 1E,F; *p* = 0.9). 

### 3.4. Respiratory Quotient (RQ)

There was no effect of day, sex, or oral contraceptive use on baseline RQ (Table 3), nor was there any significant difference in RQ response between any of the test meals (see supplementary Figure S1).

### 3.5. Visual Analog Scale (VAS) Ratings

Within each subject group, no differences in VAS ratings were observed at baseline between test days. On average men reported slightly, but significantly, less fullness at baseline than women (*p* = 0.04; Table 3), although no such differences were found in desire to eat or prospective food consumption. As expected, changes in all three VAS ratings (increase in fullness and decreases in desire to eat and prospective food consumption) were observed after each meal. However, there were no significant between-meal differences in these ratings at any time-point, nor when integrated over the three hour postprandial period.

**Figure 2:**
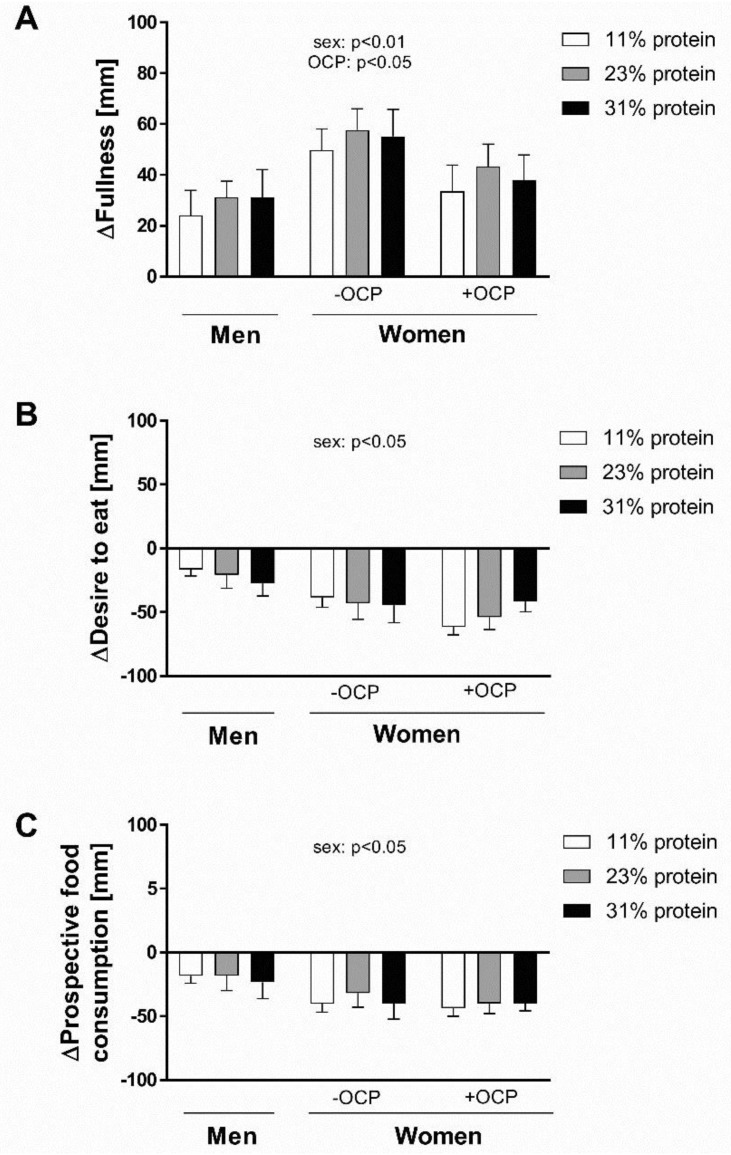
Change in visual analog scale (VAS) ratings of (**A**) fullness, (**B**) desire to eat, and (**C**) how much food the participants thought they could eat (prospective food consumption) after ingestion of an isocaloric meal containing one of three meal protein levels (11, 23, and 31% total energy as protein) relative to baseline (fasted) ratings. Values are mean ± SEM. OCP: oral contraceptive pill; ns: not significant.

Comparison across subject groups and across meals at baseline (fasting state) indicates an effect of sex for VAS ratings of fullness only (*p* < 0.05), with men scoring higher levels of fullness at baseline than women. However, analysis of the changes in VAS ratings during the postprandial period (Figure 2) indicate (i) a significant effect of sex for all three VAS ratings, with women showing a greater effect than men, and also (ii) women not taking the pill showing a significantly greater VAS ratings of postprandial fullness than either men or women taking pill (*p* < 0.05).

## 4. Discussion

In the present study we investigated the dose–response relationship between meal protein content and resting EE, respiratory quotient, and satiety in healthy young men and women, in response to three different levels of protein intake (11, 23, and 31% kcal protein), in line with the range of high protein diets often investigated for body weight regulation and maintenance. As no previous effects of the menstrual cycle on the thermogenic responses to protein meals were observed [5], this study was conducted only in the follicular phase in the female subjects. We hypothesised that resting EE and satiety would increase in a stepwise fashion in response to increasing dietary protein intake. Indeed, our results agreed with this hypothesis in men, with significant dose–response increases in EE with increasing protein dose observed in men. However, in women without OCP, the 23% protein meal increased EE above the level observed following the 11% protein meal (*p* < 0.001), but a further increase in EE between the 23% and the 31% protein meal was not observed. Strikingly, in line with our previous study [5], no effect of meal protein content was seen in women taking the OCP, with no significant difference in response between any of the test meals, representing a three-fold range of meal protein content. The mechanisms underlying this single-level response (i.e., thermogenic inflexibility) across such a wide range of protein intakes remains to be studied. However, coupled with the lack of differential response to the two higher doses in women not taking the OCP, it would appear that a theoretical metabolic ceiling may be reached, particularly in women taking the OCP. As a result, it could be hypothesised that the protein-induced thermogenic response is operating to its maximum level above a certain threshold of exogenous protein intake. The potential mechanisms involved may be related to the control of protein flux and protein turnover or its two components; namely, whole-body protein synthesis (expensive in ATP need), and whole-body protein breakdown. Both components are being increased acutely following a high protein meal, and also following prolonged feeding with a high protein diet [13]. Another metabolic process, costly in terms of ATP need, is that of liver gluconeogenesis—de novo synthesis of glucose from amino acid precursors released through increased protein breakdown. However, this process is markedly stimulated if the amount of exogenous CHO is low [14]. Further exploration of our hypothesis of a putative “physiological ceiling” would require more in-depth research by non-invasive techniques (such as the use of stable isotopes) to investigate protein metabolism and turnover. In particular, to investigate the dynamic regulation of exogenous protein utilisation in terms of changes in whole-body protein synthesis in relation to changes in whole-body protein breakdown. Although the literature is very scarce on this issue, we suspect that the hormonal changes induced by OCP are also susceptible to influence amino-acid metabolism [14]. 

In Western countries, the majority of women habitually consume a diet containing approximately 15% total energy as protein [15], and as such intervention strategies aimed at stimulating diet-induced thermogenesis through a high-protein diet could be challenged, as most of the benefit in terms of energy expenditure is already operating. Similarly, it is currently unclear if the homogenous level of response observed in women taking the OCP represents a caloric response, insensitive to meal protein content, or if there is a lower threshold of meal protein content above which the “all-or-nothing” response is stimulated. These questions warrant further investigation, particularly in light of (i) rates of female obesity exceeding those of men across all regions of the globe [16]; (ii) sex disparities in the effectiveness of current intervention strategies [17]; and (iii) the commonplace usage of the exogenous female sex hormones (e.g., for contraception or replacement therapy) [18,19,20,21].

In order to investigate the effect of increasing protein meal content on appetite and satiety, we also utilized visual analogue scale (VAS) ratings of fullness, desire to eat, and prospective of food consumption. These data reveal (i) a significant effect of sex for all three VAS ratings, with women showing a greater effect than men, and also (ii) women without OCP showing a significantly greater mean VAS rating for fullness than men or women taking the OCP. Despite several studies showing a greater satiety effect after the consumption of a high protein meal than after a normal protein meal [22], the question remains as to why the satiety ratings did not correspond to the energy expenditure data. One possible explanation, particularly in terms of the difference noted between men and women, was that the meal size (volume and caloric content) was fixed, and not adjusted according to the body size or composition of the subject. As such the calorie load was greater in the women than the men on a body size basis. Another possible reason is that the VAS scale is a subjective approach and the subject may consciously or subconsciously choose to override their appetite cues when filling in the scale. The physiologic responses are not always in line with perceived satiety related feelings, indicating that the regulation of appetite is a complex process in which different mechanism may play a role and no one factor can be held responsible [23]. Therefore, the difference observed between women taking the OCP and not taking the OCP warrants further investigation as these women were of a similar body size and body composition, indicating an effect beyond that of caloric load alone. In particular, to determine whether these results are due to a direct effect of the exogenous hormones contained within the OCP, or to the suppression of endogenous hormonal cycling, the latter having already been shown to influence energy and macronutrient intake [24].

### Study Limitations

As mentioned above, the caloric content and volume of the meal was fixed. Perhaps as a result of this, some subjects anecdotally found the highest protein level difficult to eat because of strong feelings of satiety. This problem is often addressed by the use of high protein meal replacements (e.g., “protein shakes”); however, it remains to be investigated as to whether the metabolic response to this form of protein intake is directly comparable to that of a mixed meal (more representative of daily-life). Given recognition that different protein types can exert different postprandial metabolic effects [25], caution should also be used when extrapolating the results of our study (using dairy protein) to high protein meals in general. For example, casein and whey differ not only in amino acid composition but also in absorption and digestion rates, with casein being a “slow” protein and whey being a “fast” protein, all of which may be important factors for protein-stimulated metabolic effects and thermogenesis. However, although one study has shown that whey protein stimulated thermogenesis to a greater extent than casein and soy protein [25], other studies have been inconclusive [25].

Additionally (i) although all subjects in the present study took the combined monophasic contraceptive pill, OCP formulations differed in terms of ethinyl estradiol concentration and progestin type and dose, and our results do not necessarily generalize to other OCP formulations; and (ii) a priori power calculations were only conducted for energy expenditure and not for RQ or the VAS variables, therefore power to detect differences in these variables between groups may have been limited. 

Finally, the demonstration here of differences in protein-induced thermogenesis due to OCP represent differential responses in energy expenditure to a single meal. Whether these differences would cumulate following multiple meals over the day so as to impact significantly on daily 24-h energy expenditure will need to be addressed in future studies. It should be noted that even small differences in daily energy expenditure (whether resulting from changes in BMR, in diet-induced thermogenesis or in non-resting energy expenditure) have the potential, when cumulated over months and years, to have an important impact on body weight regulation and in the development or prevention of obesity [25].

## 5. Conclusions

With approximately three quarters of the day routinely spent in the postprandial state, differences in diet-induced thermogenesis could have a significant impact on overall daily energy expenditure. Whilst increasing meal protein content may present a potential means to optimise or enhance postprandial energy expenditure, as indicated by our findings above, careful consideration needs to be paid to factors that may influence an individual’s metabolic response to such a dietary intervention. This study revealed a new, previously unknown finding in women taking the OCP, namely that the postprandial thermogenic response of the test meal was inflexible to a wide range of protein content, the underlying mechanisms of which warrant further investigations. Finally, our observed negative effect of the OCP on dynamic protein metabolism in women adds to the list of noted, but often overlooked, side effects of the OCP on nutritional metabolism, which includes well-known effects on vitamin (B2, B12, folate), mineral and trace element (magnesium, zinc) metabolism. With the prevalent use of OCPs by women of child-bearing age, such effects merit more attention within the research and clinical setting alike. 

## Figures and Tables

**Table 1:** Test meal composition (calculated) as a load for assessing postprandial thermogenesis.

Protein (% Total Energy)	Quantity (g)					Energy (kcal)			Ratio Fat: CHO
	Rolled Oats	Ground Almonds	Yoghurt ^1^	Double Cream ^2^	Protein Supplement ^3^	Protein	Fat	CHO ^4^	
11%	80	6	180	20	0	73	216	306	0.71
23%	62	12	190	13	8	137	189	267	0.71
31%	50	16	200	4	20	182	171	243	0.71

^1^ Yoghurt (11%: M-Classic Blueberry, Migros, Switzerland; 23% and 31%: YoQua Blueberry, Emmi, Switzerland); ^2^ Double Cream (Double crème de la Gruyère, Migros, Switzerland); ^3^ Protein Supplement (Protifar, Nutricia, Schiphol, the Netherlands); ^4^ CHO, carbohydrate.

**Table 2:** Participant characteristics (mean ± SEM).

	Men	Women		*p*-Value	
		-OCP ^3^	+OCP ^3^	Men v -OCP	-OCP v +OCP ^3^
*n*	7	7	7		
Age (year)	23.0 ± 0.6	24.4 ± 0.9	22.6 ± 0.6	0.6	0.12
Weight (kg)	76.1 ± 2.3	61.5 ± 2.3	59.2 ± 2.0	**< 0.001**	0.48
Height (cm)	177 ± 1.5	164 ± 2.3	168 ± 1.6	**< 0.001**	0.21
BMI ^1^ (kg/m^2^)	24.3 ± 0.6	22.8 ± 0.7	21.0 ± 0.6	**0.01**	0.08
FFM ^2^ (kg)	64.0 ± 2.0	44.5 ± 1.3	44.5 ± 1.6	**< 0.001**	0.97
%Fat	15.6 ± 2.9	26.8 ± 2.1	24.9 ± 1.9	**< 0.005**	0.52

^1^ BMI: Body mass index; ^2^ FFM: Fat-free mass; ^3^ OCP: Oral contraceptive pill.

**Table 3:** Baseline (fasted) parameters (3 day mean ± SEM).

	Men	Women		*p*-Value	
		-OCP ^3^	+OCP ^3^	Men v -OCP	-OCP v +OCP^3^
**Indirect Calorimetry**					
EE (kcal/min) ^1^	1.26 ± 0.05	0.90 ± 0.03	0.89 ± 0.03	**< 0.001**	0.74
RQ ^2^	0.809 ± 0.01	0.823 ± 0.02	0.824 ± 0.02	0.42	0.98
**Visual Analog Scales**					
Fullness (mm) ^4^	23 ± 6	12 ± 3	11 ± 4	**0.04**	0.87
Desire to eat (mm) ^4^	64 ± 5	62 ± 8	69 ± 6	0.83	0.53
Prospective food consumption (mm) ^4^	59 ± 8	64 ± 7	64 ± 6	0.52	0.95

^1^ EE: Energy Expenditure; ^2^ RQ: Respiratory quotient; ^3^ OCP: Oral contraceptive pill; ^4^ range of visual analog scale = 0–100 mm.

## Supplementary Materials

The following are available online at https://www.mdpi.com/2072-6643/11/7/1599/s1, Figure S1: Change in resting respiratory quotient (ΔRQ) following ingestion of each of three isocaloric meals containing different protein levels (11, 23, and 31% total energy as protein). Values are mean ± SEM. OCP: oral contraceptive pill.
