# Supplementary material for: Assessment of the Dose–Response Relationship between Meal Protein Content and Postprandial Thermogenesis: Effect of Sex and the Oral Contraceptive Pill
Source: Nutrients. 2019 Jul 15;11(7):1599. doi: 10.3390/nu11071599 (PMC6682860; doi:10.3390/nu11071599)
Supplement: Supplementary file 1 [file nutrients-11-01599-s001.pdf]

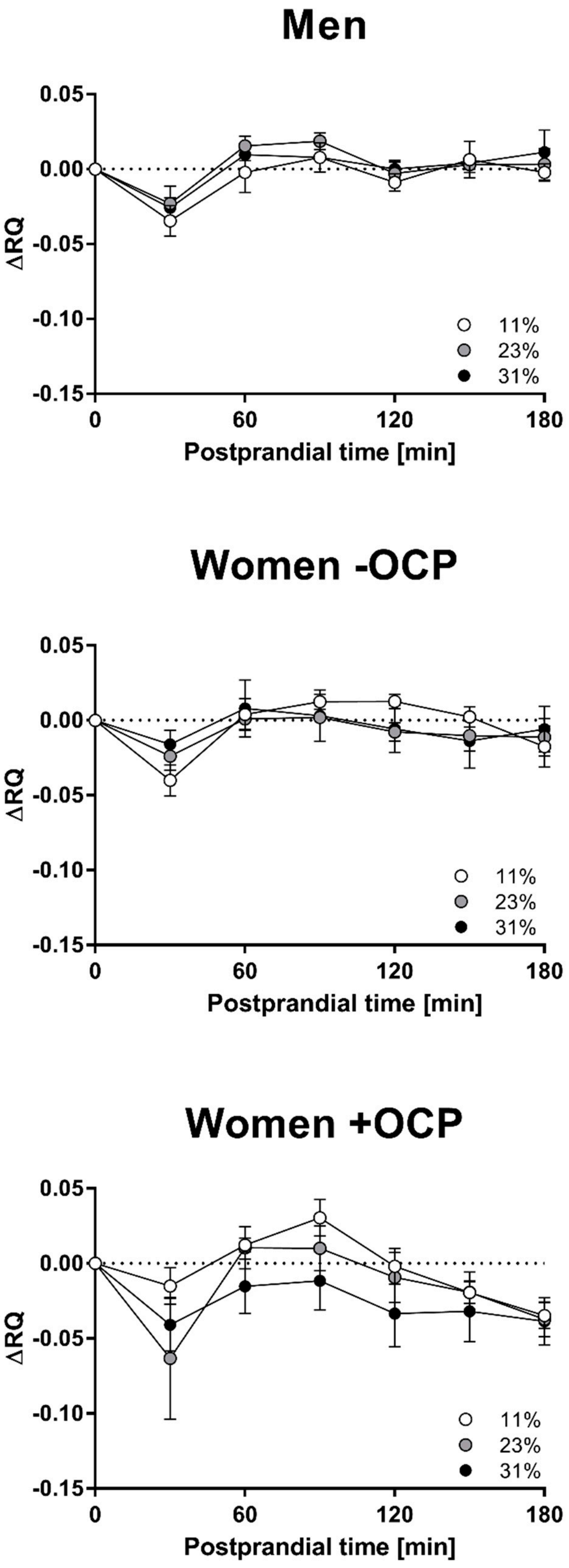

Supplementary Figure 1. Change in resting respiratory quotient (ΔRQ) following ingestion of each of three isocaloric meals containing different protein levels (11, 23, and 31% total energy as protein). Values are mean ± SEM. OCP: oral contraceptive pill.
